# Bacterial Brain Abscesses in a Patient With Transposition of the Great Arteries and Interventricular Communication

**DOI:** 10.7759/cureus.47119

**Published:** 2023-10-16

**Authors:** Ana K Gómez-Gutiérrez, Araceli Morelos-Ulibarri, Daniela Trejo-Ponce de Leon, Carla D Gomez-Flores, Eder Luna-Ceron

**Affiliations:** 1 Infectious Diseases Division, National Institute of Pediatrics, Mexico City, MEX; 2 Department of Bronchial Hyperreactivity, National Institute of Respiratory Diseases, Mexico City, MEX; 3 Department of Clinical Sciences, Tecnológico de Monterrey, Mexico City, MEX; 4 Cardiology Division, National Institute of Pediatrics, Mexico City, MEX; 5 Department of Internal Medicine, Texas Tech University Health Sciences Center, Paul L. Foster School of Medicine, El Paso, USA; 6 Laboratory of Cardiovascular Medicine and Metabolomics, Hospital Zambrano Hellion, Monterrey, MEX

**Keywords:** dental caries, neuroinfection, levo-transposition of great arteries, cyanotic congenital heart disease, brain abscess

## Abstract

Brain abscesses are localized infections in the brain's parenchyma, characterized by inflammation, pus formation, and the development of a surrounding capsule. These lesions typically occur due to underlying factors such as immunosuppression, ear and sinus infections, and contamination during neurosurgery. While brain abscesses are a life-threatening complication of cyanotic heart defects, they are rarely reported, with only sporadic cases previously documented. This article presents the case of an eight-year-old male patient with an uncorrected transposition of the great arteries, who was evaluated for symptoms including headache, fever, and neurological focalization. Diagnostic imaging revealed three lesions consistent with brain abscesses. Furthermore, the causal agents were identified as *Streptococcus intermedius* and *Fusobacterium* spp., representing oral microorganisms. Additionally, the patient exhibited poor oral hygiene and dental caries in multiple teeth. This article discusses and integrates the possible pathophysiological mechanisms that allowed a localized dental infection to spread hematogenously and cause brain abscesses in this patient. Prompt management of the infectious source is crucial to prevent a poor prognosis associated with brain abscesses. Therefore, this case emphasizes the importance of regular dental assessments and thromboprophylaxis for patients with underlying cardiomyopathies that cause right-to-left shunting to prevent potential complications.

## Introduction

Brain abscesses (BAs) are characterized as localized infections within the brain parenchyma involving inflammation, pus formation, and the subsequent development of a surrounding capsule [1]. Some authors also require the exclusion of cancerous or lymphomatous cells to confirm the infectious origin [2]. The estimated annual incidence of BA ranges from 0.3 to 1.88 cases per 100,000 individuals [3,4]. Incidence rates are higher in patients with underlying immunosuppression [2]. BAs are more commonly diagnosed in males [2,4,5], while the average age of patients varies widely among studies [4,6]. Pediatric cases account for less than 16.4% of BA cases, indicating their rarity compared to adult cases [7].

There are three main mechanisms through which pathogens contribute to BA formation: hematogenous dissemination, direct dissemination from infectious foci, and direct inoculation through neurosurgery or trauma [1]. The primary mechanism involved in an infection depends on the underlying predisposing factors of each patient. Direct dissemination accounts for 25-50% of BA cases [2,3,6]. The frontal lobe, the most frequent location for BA, is commonly associated with direct dissemination from a contiguous source [4,8]. Hematogenous dissemination is often associated with underlying chronic conditions such as cardiac or pulmonary diseases, or distant foci of infection, and represents 15-30% of cases [2,3,6]. Additionally, in 20-30% of cases, the origin or initial infectious source remains unidentified [2].

Several factors influence the specific presentation of BA, including predisposing factors, patient age, immune status, and prior antibiotic use. Multicenter clinical studies have shown that predisposing factors are more common in BA cases compared to matched controls, occurring in 56-86% of cases [3,6,9,10]. The most prevalent acute risk factors in children are sinus and ear infections, neurosurgery, and head trauma [5,9]; notably, meningitis plays a predominant role in infants [9]. Importantly, a significant association has been observed between BA and congenital heart disease [7,10]. Although the link between congenital cardiac defects and BA is clear, it is primarily attributed to the presence of pulmonary circulation shunts and arteriovenous fistulas [7,10]. However, the occurrence of underlying cyanotic congenital defects remains rare [11,12]. In this article, we present a case of a pediatric patient with a previous history of uncorrected transposition of the great arteries (TGA) who developed BA due to the formation of paradoxical septic thrombi.

## Case presentation

**Figure 1:**
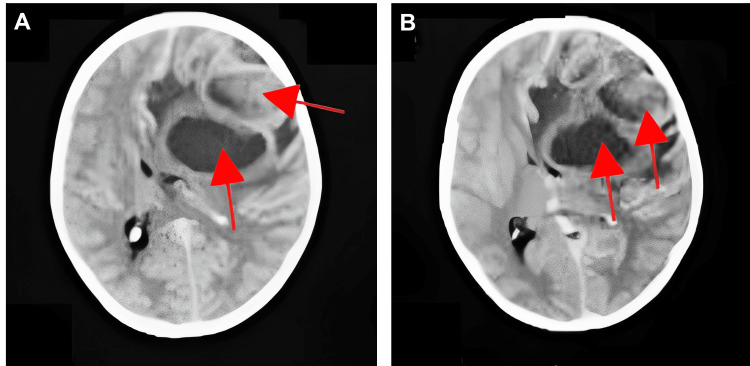
Representative slides (A-B) of cranial computed tomography revealing hypodense masses in the temporal and frontal lobes compatible with brain abscesses. Red arrows show three well-defined hypodense masses (17-20 Hounsfield units) in the frontal-parietal region with hyperdense halos with an average length of 5.3 mm and surrounding edema, which displaces the midline.

We present the case of an eight-year-old male patient with a previous diagnosis of TGA at six months of age, who presented to our emergency department with a three-day history of severe headache and fever reaching 39°C. The patient's mother also reported a loss of appetite and several episodes of nausea and vomiting. Prior to visiting our center, the patient had been evaluated by a general pediatrician who ordered a cranial computed tomography (CT) scan and a complete blood count (CBC). The CT scan revealed the presence of three hypodense masses with perilesional edema and mass effect, consistent with BAs (Figure 1). The CBC results at the time indicated polycythemia and leukocytosis. The patient was initiated on vancomycin and ceftriaxone for antibiotic therapy. Given the findings, the patient was referred to our center for further management.

During the initial pediatric evaluation, the patient appeared somnolent and irritable but without respiratory distress. The patient's skin color and turgor were normal, and his airway was permeable. His respiratory rate was 22 respirations per minute (rpm), oxygen saturation was 82% on room air, and pulse rate was 61 beats per minute (bpm). His blood pressure was within the normal range (91/59 mmHg, percentile 52), capillary filling time was two seconds, and he had a fever (38.5°C). Neurological examination revealed pupils equally round and reactive to light, as well as facial asymmetry on the right side. Examination of the oral cavity showed dental caries in multiple teeth and signs of poor oral hygiene. Cardiac examination revealed low-intensity S1 and S2 heart sounds and a holosystolic murmur audible in all cardiac fields. The patient exhibited hyperreflexia in the right lower limb with preserved strength and sensitivity. Peripheral cyanosis and digital clubbing were observed in both hands. The rest of the physical examination revealed normal findings.

For acute management, the patient was administered 1 liter per minute of supplemental oxygen for ventilatory support, but there was no significant improvement in oxygen saturation (85%) due to his underlying cardiopathy. The antibiotic therapy was adjusted to include metronidazole for coverage against anaerobic bacteria. Neurosurgery was consulted, and the patient was scheduled for surgical drainage of the BAs. The pediatric cardiology service was also consulted for echocardiography to guide the management of the patient's underlying cardiopathy. The laboratory results obtained upon admission are presented in Table 1.

**Table 1:** Laboratory investigations performed on the admission day. Results are presented in parameters and their corresponding units. IU: international units.

Parameter (units)	Result
Hemoglobin (g/l)	21.1
Hematocrit (%)	65.5
Leucocytes (cells/mm^3^)	12,300
Neutrophils (cells/mm^3^)	9,900
Lymphocytes (cells/mm^3^)	1,800
Platelets (cells/mm^3^)	322,000
Prothrombin time (seconds)	15.3
International normalized ratio (IU)	1,37

The day after admission, the patient underwent BA drainage under general anesthesia. During the procedure, the surgical team successfully drained 120 ml of purulent material, which was sent for culture analysis. The surgery was completed without complications, and the patient was immediately transferred to the neurological intensive care unit (NICU) for postoperative care. Within the NICU, the patient remained hemodynamically stable without the need for aminergic or diuretic support. Additionally, the patient remained afebrile after the surgery. The cardiology department conducted an electrocardiogram (EKG) and thoracic echocardiography (TE) as part of the diagnostic evaluation for the underlying cardiopathy. The TE revealed a 45 mm interventricular communication (IVC) with resulting right ventricular (RV) hypertrophy, without evidence of endocarditis. The EKG showed signs suggestive of RV hypertrophy without any other significant findings. While in the NICU, the patient received an assessment from the dental department, which revealed generalized gingivitis, incomplete dentition for his age, and multiple infectious foci in the oral cavity.

**Figure 2:**
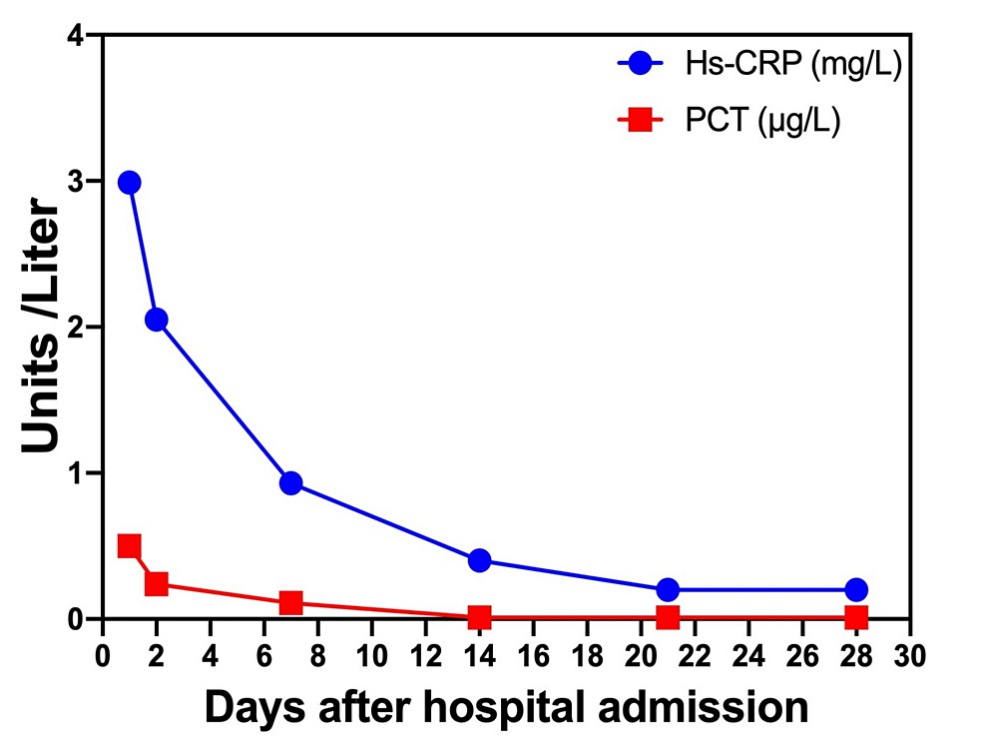
Acute phase reactant tendency over the course of the patient's hospitalization. Data are plotted in µg/L for PCT and mg/L for Hs-CRP. Hs-CRP: high-sensitivity C-reactive protein; PCT: procalcitonin.

The purulent material obtained from the drainage was subjected to culture, which identified the presence of gram-positive cocci and gram-negative bacilli populations. Further characterization of the isolates confirmed the presence of *Streptococcus intermedius* and *Fusobacterium* spp. The patient's antibiotic therapy was continued successfully for a duration of 42 days. Throughout this period, the patient remained afebrile, and the laboratory results of acute phase reactants exhibited a declining trend, as illustrated in Figure 2. Antibiotic treatment was continued until the patient's discharge.

On the fifth day post surgery, the patient's buprenorphine medication for pain management was carefully discontinued. However, the patient continued to receive levetiracetam for seizure prophylaxis. Throughout the hospital stay, the patient did not exhibit any abnormal movements. To address the risk of developing new septic emboli, the case was discussed with the hematology team, resulting in the implementation of a thromboprophylaxis plan. The patient was initiated on enoxaparin. Stomatology services were involved in the patient's care during the hospital stay, providing comprehensive guidance and education to prevent new dental lesions that could potentially serve as sources of infection. Following an uncomplicated recovery, the patient was discharged and referred to the cardiology department for the management of his congenital cardiac defect.

## Discussion

We present the case of an infant who presented with an acute episode of febrile headache, neurological focalization, and vomiting, which collectively suggested the presence of a neuro-infection. BA was our primary differential diagnosis in this case, as it can lead to increased intracranial pressure and manifest with this specific triad of symptoms [1]. The classic triad of BA consists of headache, fever, and focal neurological findings [1,3], with papilledema and seizures occurring less frequently [7].

On CT imaging, BA typically exhibits a hyperdense external and internal rim with uniform thickness surrounding a center of low attenuation material, indicating central necrosis within the lesion [13,14]. The lesions are also accompanied by low attenuation corresponding to vasogenic edema [14]. In some cases, the extension of the infective foci can lead to ventriculitis, which can be observed as ependymal hyperdensity [13,14]. In our patient's case, we observed the classical lesions with central attenuation and peripheral rim hyperdensity, primarily affecting the temporal and parietal lobes. While magnetic resonance imaging (MRI) is more sensitive, the presence of these characteristic lesions, along with the clinical triad for BA, strongly suggested a presumptive diagnosis of BA in this patient. It is worth noting that many population studies include cranial imaging confirmation as an inclusion criterion, irrespective of the suspected etiology [3]. CT is the most used initial imaging modality in almost 88% of pediatric cases [3]. These findings suggest that cases where the initial CT imaging misses the diagnosis may be identified with subsequent MRI scans [3,4].

The infectious agents associated with BA are similar in children and adults and depend on the underlying mechanism of infection [2]. Bacteria are the most commonly isolated infectious agents, while mycobacteria, protozoa, helminths, and fungi are less frequently involved [3,15]. *Streptococcus* spp. (particularly *S. anginosus*) are the most cultured microorganisms in BA, accounting for approximately one-third of cases. *Staphylococcus* spp. are the second most reported microorganisms [3,15], often associated with previous penetrating head injury or neurosurgery [3,8,15]. Other notable agents, regardless of origin, include gram-negative enteric bacteria and *Haemophilus* spp. [3,9]. Polymicrobial infections account for up to 27% of cases [2,16]. Remarkably, our case exhibited polymicrobial growth of *Streptococcus* spp. and *Fusobacterium*, consistent with these reports.

Considering the isolation of oral microorganisms and the physical examination findings, which revealed multiple caries and poor dental hygiene in our patient, it strongly indicated that the oral cavity was the source of the infectious foci. Microorganisms commonly associated with periodontal and periapical infections include *Streptococcus* spp., *Bacteroides* spp., *Fusobacterium*, and *Peptostreptococcus* spp. [17]. Given this context, our objective was to investigate a potential cause for bacterial dissemination.

Diagnosing BA associated with odontogenic infectious sources involves specific criteria [17]. Patients must exhibit no other infectious sources, microbial evidence of pathogens typically found in perioral infections, and clinical or radiological evidence of dental infection [17]. Cultures should be obtained directly from BA lesions after drainage. In the case of our patient, all criteria were met, allowing us to focus on preventing further BA by prioritizing dental care.

TGA is a congenital cardiac defect resulting from abnormal development of the aorta and pulmonary trunk during embryogenesis [18]. This condition is characterized by the aorta arising from the right ventricle and the pulmonary trunk arising from the left ventricle in a discordant manner [18]. Consequently, this abnormality creates two parallel circulation circuits, leading to the delivery of deoxygenated blood to the systemic circulation, rendering it incompatible with life [19]. However, patients with concomitant patent ductus arteriosus or ventricular septal defects can survive, as they allow the mixing of oxygen-rich blood from the opposing circulation circuit [18]. Typically, survival rates are low in patients with uncorrected TGA, with mortality rates reaching approximately 30% in the first week of life and as high as 90% by the end of the first year [18]. Although survival facilitated by a communication defect is not without complications, such as the development of polycythemia, hyperviscosity syndrome, congestive heart failure, ventricular arrhythmias, and delayed developmental milestones [18]. The occurrence of BA, a life-threatening complication of TGA, is not commonly reported, with sporadic cases having been previously published [11]. Other cyanotic cardiac defects, such as tetralogy of Fallot (TOF), which have a higher prevalence, also show BA development as a rare complication, occurring in approximately 5% to 18% of patients [11,12]. Communication defects in these cases can facilitate the hematogenous dissemination of distant infections, putting patients with these congenital diseases at particular risk for such events [11,12]. This mechanism can explain the spreading of dental infection into the development of BA in our patient.

Surgical intervention followed by antibiotic therapy is the established standard treatment for BA, regardless of its origin [4,16]. The availability of newer neuronavigation systems has significantly reduced complications associated with surgery [9]. Similarly, in our case, surgical intervention was the primary approach. Empiric treatment for BA typically involves a third-generation cephalosporin with metronidazole, which effectively targets the causative gram-positive and gram-negative bacteria, as well as anaerobes in approximately 83% of cases [5,17]. In line with these recommendations, we followed this treatment regimen and added vancomycin to cover a possible co-infection involving *Staphylococcus* [1].

However, it should be noted that Felsenstein et al. suggested that cephalosporins alone could be sufficient in cases involving congenital heart disease or meningitis [5]. Nevertheless, the increasing rates of observed resistance may challenge this intervention. Additionally, the duration of antibiotic therapy varies significantly, with reported durations ranging from five to 176 days, although the most common duration is six weeks [5,9]. In our patient's case, antibiotic therapy was completed prior to discharge after six weeks of treatment.

BA development is a potentially life-threatening complication of congenital cardiac defects, with reported mortality rates ranging from 10% to 24% in various studies [10]. However, over the past few decades, case fatality rates have improved, with up to 70% of patients achieving favorable recoveries following adequate treatment [4]. Nevertheless, long-term sequelae can be expected, including new-onset epilepsy in up to 11% of cases and residual neurological deficits in 7.9% of survivors [20]. Generally, worse outcomes are associated with younger age (less than five years) and a Glasgow Coma Scale (GCS) score of eight or less at presentation [4,5,20]. In our patient's case, none of these poor prognosis predictors was observed. Nonetheless, close monitoring of his progress was maintained to prevent the development of adverse outcomes.

## Conclusions

Although BAs are less prevalent in pediatric patient populations compared to adults, it is imperative that patients suspected of BA undergo a comprehensive physical examination to identify potential risk factors for its development, including a thorough oral assessment. This clinical case contributes novel insights to the medical community, emphasizing the importance of regular dental evaluations and thromboprophylaxis for patients with underlying cardiomyopathies, particularly those predisposing to right-to-left shunts, to prevent potential systemic infectious complications.

Furthermore, this article underscores the necessity of prompt evaluation in patients exhibiting evidence of BA, with a particular focus on identifying potential sources of infection. It is crucial to note that any delay in addressing the infectious source of BA can significantly impact the patient's prognosis. This case scenario also sheds light on the mechanistic events that may underlie the infrequently reported but existing association between congenital heart disease and the development of BA.
